# *In vitro* flubendazole-induced damage to vital tissues in adult females of the filarial nematode *Brugia malayi*

**DOI:** 10.1016/j.ijpddr.2015.06.002

**Published:** 2015-07-07

**Authors:** Maeghan O'Neill, James F. Geary, Dalen W. Agnew, Charles D. Mackenzie, Timothy G. Geary

**Affiliations:** a Institute of Parasitology and Centre for Host-Parasite Interactions, McGill University, Ste-Anne-de-Bellevue, QC H9X 3V9, Canada; b Department of Pathobiology and Diagnostic Investigation, Michigan State University, East Lansing, MI 48824, USA

**Keywords:** Filariasis, Macrofilaricide, Benzimidazole, Histology, Reproduction

## Abstract

The use of a microfilaricidal drug for the control of onchocerciasis and lymphatic filariasis necessitates prolonged yearly dosing. Prospects for elimination or eradication of these diseases would be enhanced by availability of a macrofilaricidal drug. Flubendazole (FLBZ), a benzimidazole anthelmintic, is an appealing candidate macrofilaricide. FLBZ has demonstrated profound and potent macrofilaricidal effects in a number of experimental filarial rodent models and one human trial. Unfortunately, FLBZ was deemed unsatisfactory for use in mass drug administration (MDA) campaigns due to its markedly limited oral bioavailability. However, a new formulation that provided sufficient bioavailability following oral administration could render FLBZ an effective treatment for onchocerciasis and LF. This study characterized the effects of FLBZ and its reduced metabolite (FLBZ-R) on filarial nematodes *in vitro* to determine the exposure profile which results in demonstrable damage. Adult female *Brugia malayi* were exposed to varying concentrations of FLBZ or FLBZ-R (100 nM–10 μM) for up to five days, after which worms were fixed for histology. Morphological damage following exposure to FLBZ was observed prominently in the hypodermis and developing embryos at concentrations as low as 100 nM following 24 h exposure. The results indicate that damage to tissues required for reproduction and survival can be achieved at pharmacologically relevant concentrations.

## Highlights

• Detrimental effects observed in tissues required for development and survival.
• Damage occurs at concentrations ≥100 nM.
• Hypodermal and embryonic tissues exhibited the most severe damage.
• Oocytes and early morula are the most affected embryonic tissues.

## Graphical abstract

**Figure:**
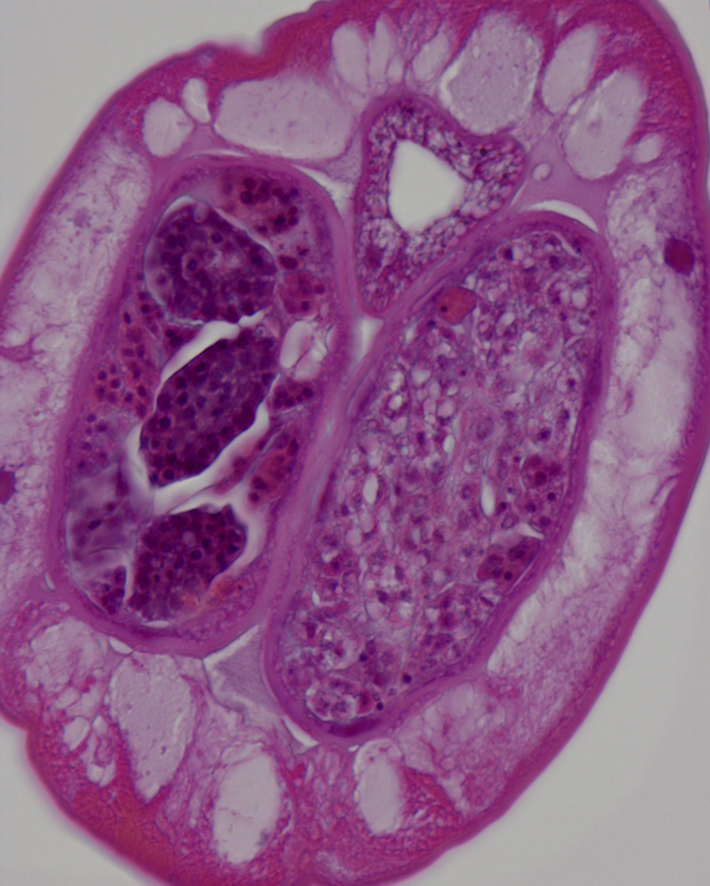

## 1 Introduction

The debilitating diseases onchocerciasis and lymphatic filariasis (LF) are major causes of long term disability and impede socioeconomic development in endemic countries (WHO, 1995; WHO, 2010). Despite the magnitude of the problem, there is optimism about prospects for the elimination of onchocerciasis (Basanez et al., 2006; Mackenzie et al., 2012) and eradication of LF, with the World Health Organization targeting it for elimination by 2020 (WHO, 2010). To increase the likelihood that this goal will be achieved, it is important to address the challenges inherent to current chemotherapeutic strategies used in these programs. The drugs employed in mass drug administration programmes are principally microfilaricidal agents, and also limit reproduction; this strategy reduces transmission and the development of pathology in onchocerciasis but necessitates annual or twice-yearly dosing for many years. An effective and safe macrofilaricide would clearly shorten the time required to reach program goals. In addition, a macrofilaricide would have the benefit of reducing pathology in LF, in which the characteristic sequelae of elephantiasis and hydrocele are initiated by adult worms residing in lymphatic vessels.

Flubendazole (FLBZ), a benzimidazole (BZ) anthelmintic, is an appealing prospective macrofilaricide for use in onchocerciasis and LF. First developed for gastrointestinal (GI) nematodes of animals, FLBZ was found to be potent and efficacious for this indication (Bradley et al., 1983). Subsequently, FLBZ was approved for the treatment of human intestinal parasites (Horton, 1990), an indication for which it is also highly effective (Yangco et al., 1981; Kan, 1983). What is most appealing in the current context is the very high macrofilaricidal efficacy attained in experimental filarial rodent models (Denham et al., 1979; Mak, 1981; Mackenzie and Geary, 2011) and in a human trial in onchocerciasis (Dominguez-Vazquez et al., 1983).

Early *in vitro* studies of BZ anthelmintic effects focused on GI nematodes. Ultrastructural observations of *Ascaris suum* 6 h following exposure to mebendazole (Borgers and DeNollin, 1975; Borgers et al., 1975) showed a loss of microtubule structures in intestinal cells. Further exposure resulted in decreased glycogen content, depletion of apical secretory granules, and accumulation of secretory granules near the Golgi, associated with swelling and disruption of microvilli (Borgers and DeNollin, 1975; Borgers et al., 1975; Atkinson et al., 1980). FLBZ-induced damage to reproductive organs of filariae has also been reported (Howells and Delves, 1985; Cárdenas et al., 2010).

Other investigators reported similar findings after FLBZ exposure in culture of *Toxocara canis* and *A. suum*, including vacuolization of the musculature, female gonadal tissue, intestine, and, to a lesser degree, the hypodermis (Hanser et al., 2003). Swelling of intestinal cell endoplasmic reticulum and complete disruption of intestinal cells occurred. Following FLBZ treatment of infected animals, loss of intestinal microtubules from cells in the GI tract of the filarial nematodes *Brugia malayi* and *Litomosoides sigmodontis* was observed, using transmission electron microscopy, when the parasites were recovered as soon as 6 h post-dosing (Franz et al., 1990). Increasingly severe damage to other tissues, including the hypodermis and reproductive tissues, was observed as time after dosing increased.

FLBZ is efficacious in humans infected with *Onchocerca volvulus* (Dominguez-Vazquez et al., 1983; Mackenzie and Geary, 2011). Recent efforts have been made to develop a new formulation of FLBZ that would enable oral dosing rather than the parenteral routes used in previous studies (Ceballos et al., 2014; Longo et al., 2014). Definition of the pharmacokinetic profiles needed for efficacy with an orally-bioavailable formulation would be facilitated by knowledge of the time-concentration exposure profiles at which FLBZ is detrimental to the survival of adult filariae. The present study examines time- and concentration-dependent morphological changes in *B. malayi* adult females caused by exposure to FLBZ *in vitro*.

## 2 Methods

### 2.1 Parasites

Adult female *B. malayi* were isolated from the peritoneal cavity of jirds (*Meriones unguiculatus*) > 120 days post-infection as described (Moreno and Geary, 2008; Bennuru et al., 2009). Briefly, recovered adult worms were washed three times with warm (37 °C) RPMI-1640 medium supplemented with 100 U/mL penicillin, 100 μg/mL streptomycin, and 0.25 μg/mL amphotericin B (Sigma–Aldrich Corp., St. Louis, MO, USA; hereafter referred to as RPMI).

Adult females were exposed to varying concentrations of FLBZ or its reduced metabolite (FLBZ-R) (10 nM, 100 nM, 1 μM, 10 μM; Epichem Pty Ltd, Murdoch, WA, Australia) over a period of 24, 48, 72, 96 or 120 h, with media changes every 24 h. FLBZ and FLBZ-R solutions were prepared by dissolving the respective drug in 100% DMSO, and added to RMPI to a final DMSO concentration of 0.1%. Control RPMI contained an equivalent percent of DMSO. Three females were cultured in 1 mL RPMI at 37 °C, 5% CO_2_ and 95% humidity in each concentration. All worms were fixed for subsequent histological analysis.

Parasite isolation and culture was conducted at the Filariasis Research Reagent Resource Center in Athens, GA, USA.

### 2.2 Assessment of parasite motility

Parasite motility was assessed visually under light microscopy. Motility was scored as either: immotile, with no motion during the observation period; slightly motile, where only twitching of the head and/or tail was observed; moderately motile, with slow sinusoidal movements; or highly motile and comparable to the drug-free control. Each sample was observed for at least one minute for scoring of motility.

### 2.3 Histological preparation

*B. malayi* were fixed in glutaraldehyde (5% in 0.1 M sodium cacodylate buffer, pH 7.2; five worms in 1 mL) for a minimum of 48 h in preparation for histological processing. Worms from each treatment were combined into groups and coiled prior to embedding in Histogel (FisherScientific), which allowed visualization of various anatomical regions in multiple worms on a single slide. Dehydration, clearing, and vacuum infiltration with paraffin were completed using a Sakura VIP tissue processor. Parasites were then embedded in paraffin with a ThermoFisher HistoCentre III embedding station. A Reichert Jung 2030 rotary microtome was used to cut 4–5 micron sections, which were dried at 56 °C for 2–24 h. Slides were stained with haematoxylin and eosin prior to examination under light microscopy (60 and 100× magnification).

### 2.4 Assessment of worm damage

**Fig. 1:**
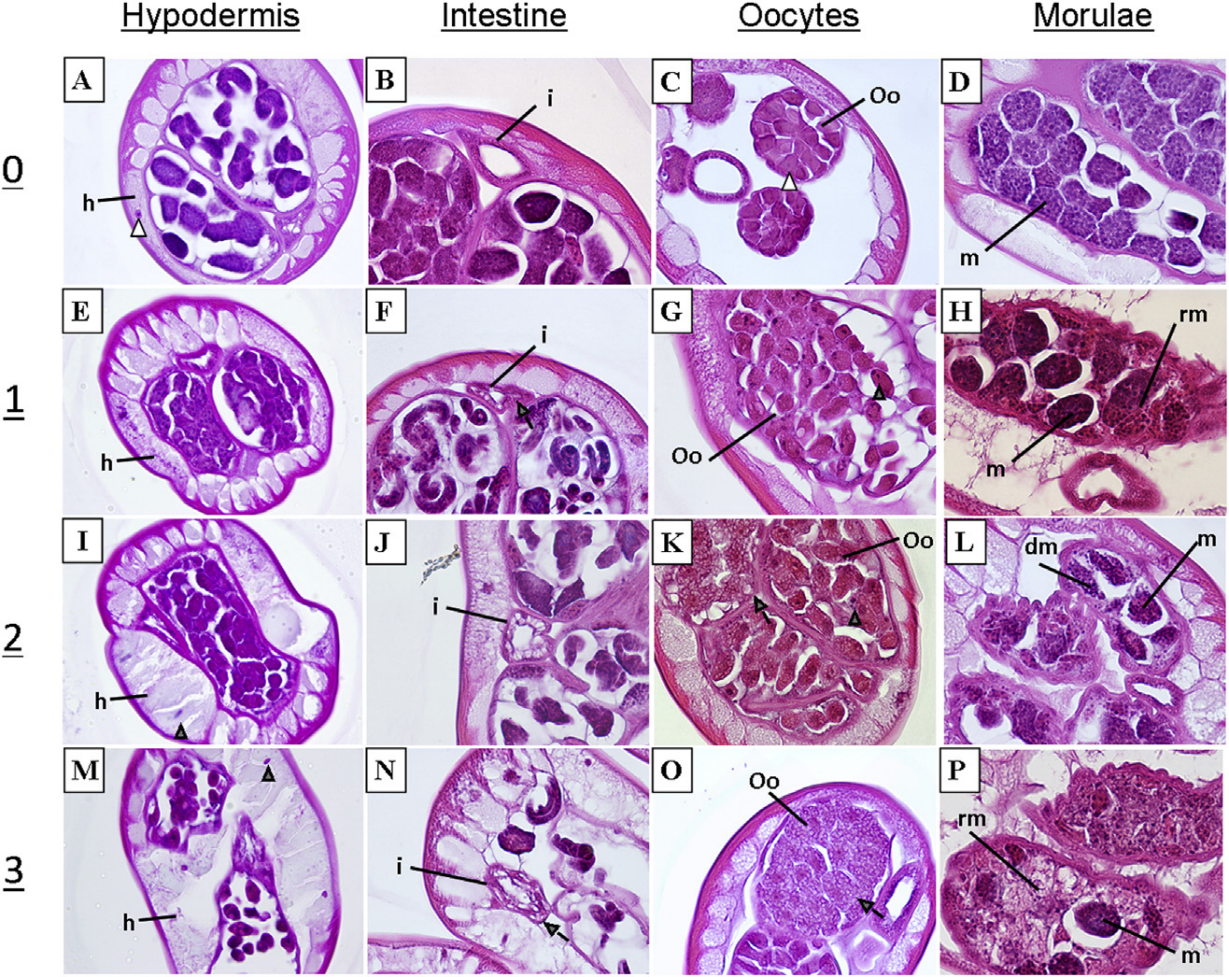
Scoring system for tissue damage observed following incubation in FLBZ (E–P) as compared to control worms (A–D). Damage was scored as mild (1), moderate (2), severe (3), or no damage (0). HYPODERMIS (h): damage was determined by observing the level of vacuolization (gray arrow) and degree of swelling in the hypodermis. Loss of integrity to the hypodermal wall was considered to be severe. Shrunken and densely staining nuclei (gray arrowhead) as compared to controls (open arrowhead) also elicited a higher damage score. INTESTINE (i): Intestinal damage was largely determined by the number, size and shape of vacuoles in the tissue. Disruption of the intestinal borders was considered to be severe damage. OOCYTES (Oo): Early embryos (including oocytes and morulae) exhibited the most damage of all the embryonic stages. Discrimination of the oocyte cell border, degree of vacuolization and nuclear abnormalities dictate the level of damage. MORULAE (m): damaged morulae exhibited loss of cellular organization. Disintegrating morulae (dm) were classified with a higher damage score. The presence of morulae remnants (rm) also factored into the score given.

Sections were assessed independently by three parasitologists familiar with filarial nematode morphology, including one board certified pathologist/parasitologist (CDM); a second board certified pathologist was also consulted in planning and developing the system (DWA). Worms from two independent experiments were examined for damage to the following tissues: body wall, including cuticle, hypodermis and longitudinal muscle; intestine; and reproductive tract, including the uterine wall and embryonic stages (classified as early [ovary, oocytes, early morulae, late morulae] or late [sausage, pretzel, microfilariae]); and pseudo-coelomic space. To aid the comparative analysis of drug-derived effects, tissues were classified into four categories of damage: no damage (0), minor (1), moderate (2), severe (3). This damage score was determined by assessing tissues for nuclear and cytoplasmic distortions, cellular size and shape, membrane integrity, accumulation of debris, and distortion of overall anatomical integrity (Fig. 1).

Two methods of analysis were performed. The first method adhered to classical techniques used by histopathologists to determine tissue damage, in which all sections on a slide were surveyed, interpreted and translated into a single damage score for each tissue type. The second method involved scoring damage in each tissue type for each worm section on a slide. These scores were then averaged for all sections on the slide to obtain the damage score.

## 3 Results

### 3.1 Quality of sections

Control worms retained well-preserved tissue structure for up to 72 h in culture. Noticeable loss of normal condition occurred in control groups after 96 h, seen as an increase in vacuolization in the intestine and hypodermis. A high degree of variability in this latter morphological change was observed among worms in the same treatment group, as well as along the length of an individual worm. Two independent experiments were conducted in an attempt to reduce this variability. Multiple transverse sections were assessed to enable estimation of the proportion of the specimen which was damaged. Samples from the 96 and 120 h incubations were excluded from further analyses to eliminate the influence of this loss of condition, presumably resulting from the effects of culture, as a variable.

Sections that displayed effects which were clearly consequences of histological processing and not due to drug exposure, such as scored sections, abnormally broken tissue structures, were excluded from analysis.

Limited damage was observed following exposure to 10 nM flubendazole; therefore, this concentration was not included in further experiments or in those with reduced flubendazole.

Control worms were exposed to 0.1% DMSO for 24, 48, or 72 h; no samples were taken for analysis immediately after removal from the jird.

### 3.2 Assessment of damage

The overall damage score for all tissues at each time and concentration using the survey method and the individual section method are shown in Tables 1A and 2A and Tables 1B and 2B respectively.

The individual section method returned higher damage scores than the survey method and detected damage that was not scored with the survey method. However, the overall conclusions reached by either method were the same. While the survey method is less labor intensive, it may miss some of the minor changes resulting from drug exposure. A shortcoming of the individual section method centers on processing of the slides. It is assumed that the sections on each slide are representative of damage occurring along the length of the worm, and that a representative number of sections from each worm in the group is assessed.

### 3.3 FLBZ-induced damage

**Fig. 2:**
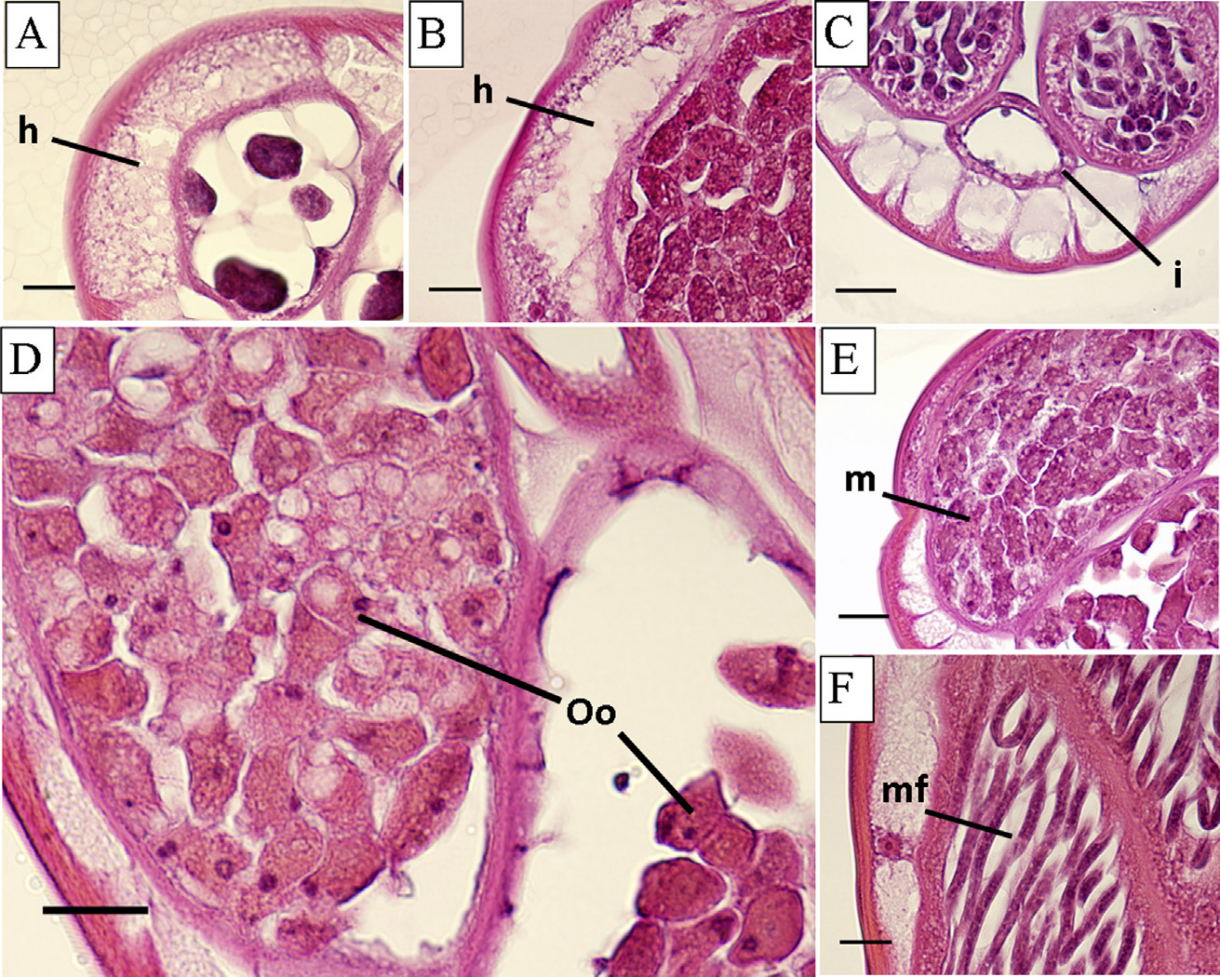
Representative damage observed following 24 h incubation in flubendazole. A and B. Hypodermis (h), 100 nM; C. Intestine (i), 100 nM; D. Oocytes (Oo), 100 nM; E. Early morulae (m), 1 μM; F. Microfilariae (mf), 10 μM (48 h). Scale bars are 15 μm.

Drug-induced damage was observed in reproductive and hypodermal tissues, and in the intestine to a lesser degree (Table 1, Fig. 2). The damage observed in the hypodermis was predominantly tissue swelling and nuclear abnormalities, including shrinking. Moderate damage to the hypodermis was visible after 24 h in 100 nM FLBZ (Fig. 2A and B, Table 1). Increased vacuolization and expansion of the hypodermis was observed in treated vs. control parasites. In some cases, complete disruption of the hypodermal membrane was observed (Fig. 2B). Damage to intestinal cells was highly variable. In some cases, intestinal cells were highly vacuolated compared to control; however, the intestinal walls remained intact (Fig. 2C).

Vacuolization of early embryos within female gonads was the first observable damage to reproductive tissues of treated worms (Fig. 2D and E). Early developmental stages exhibited more damage than later stages; oocytes and early morulae (2–12 cells) were the most severely damaged. Degradation of early embryos was observed following incubation in 100 nM FLBZ, with evident loss of cellular integrity (Fig. 2E). There was little to no damage to later developing stages. Sausage forms exhibited damage similar to what was seen in morulae, however, the pretzel stage and stretched microfilariae (Fig. 2F) remained unchanged.

**Fig. 3:**
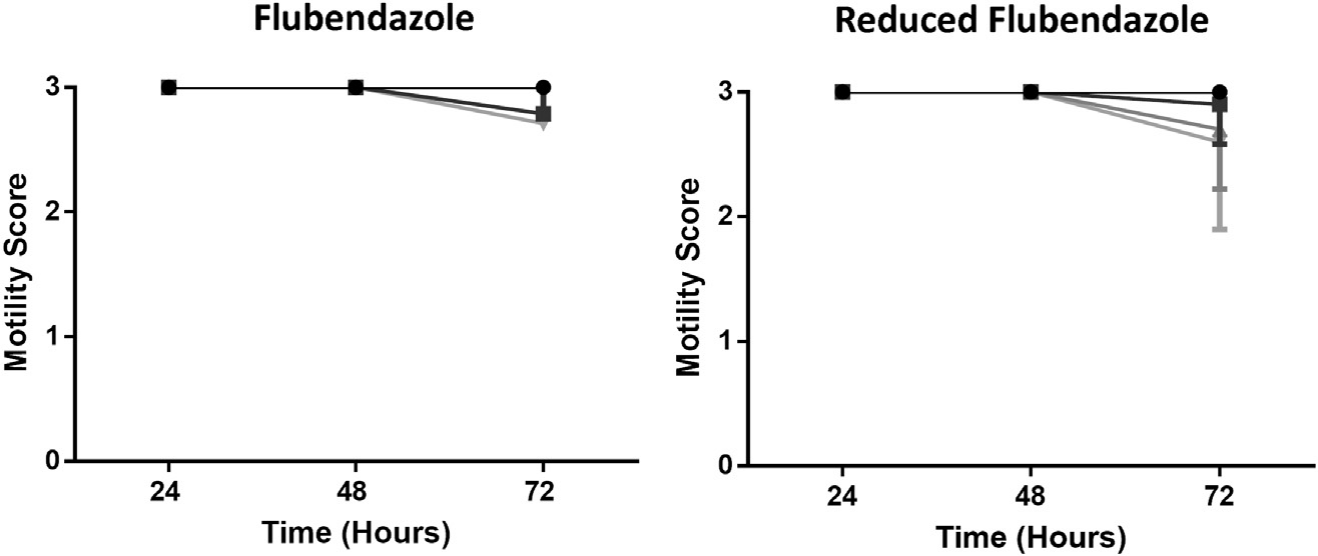
Mean motility (±SD) after exposure to flubendazole or reduced flubendazole; 10 μM (light gray triangles), 1 μM (gray triangles), 100 nM (gray squares), control (0.1%DMSO; black circles).

FLBZ had no effect on worm motility (Fig. 3).

### 3.4 Effects of reduced flubendazole

Effects of FLBZ-R were similar to those observed in FLBZ treated worms. Damage was observed in the hypodermis following exposure to 1 μM FLBZ-R for 72 h (Table 2). Moderate damage to the intestinal epithelium required longer incubations in FLBZ-R (Tables 1 and 2). Reproductive tissues were damaged following incubation in 100 nM FLBZ-R, similarly to FLBZ. As was found with FLBZ, FLBZ-R-induced damaged presented as increased vacuolization, nuclear abnormalities and disruption of cellular integrity.

FLBZ-R had no effect on worm motility (Fig. 3).

## 4 Discussion

In this study, morphological damage following *in vitro* exposure to FLBZ was observed prominently in the female reproductive tissue and hypodermis, and to a lesser extent in the intestine. Previous studies of ultrastructural changes associated with BZ exposure focused on events occurring in parasite intestinal cells all used electron microscopy, rather than the light microscopic approach used in this study. Ultrastructural alterations were detected as soon as 6 h of exposure (Borgers and DeNollin, 1975; Borgers et al., 1975; Atkinson et al., 1980), including decreased glycogen content, loss of apical secretory granules and occurrence of multiple granules near the Golgi apparatus, ultimately leading to swelling and complete disruption of microvilli (Borgers and DeNollin, 1975; Borgers et al., 1975; Atkinson et al., 1980; Comley, 1980; Zintz and Frank, 1981; Jasmer et al., 2000; Hanser et al., 2003). Although these studies described drastic morphological changes elicited by BZs in intestinal cells, they focused on GI nematodes, and the effects may not be the same for filariae. Franz et al. (1990) found that, while FLBZ exposure resulted in disappearance of microtubules from intestinal cells of *B. malayi* and *Litomosoides carinii*, the cells remained intact for up to 6 days post-exposure. It was suggested that this result reflects the limited use of intestinal cells for nutrient uptake by adult filariae. In the closely related parasite *Brugia pahangi*, transcuticular nutrient acquisition occurs in microfilariae and adult worms, yet the adults are also able to acquire nutrients orally (Howells and Chen, 1981). Lawrence and Simpson (1973) found the pharynx of *B. pahangi* microfilariae to be incompletely developed, suggesting that they acquire nutrients only across the cuticle. The capacity to acquire nutrients transcuticularly suggests that the hypodermis should exhibit more extensive damage than the intestine following FLBZ exposure. In this study, there was observable damage to the hypodermis (Fig. 1E, I and M), presenting as increased vacuolization, swelling and nuclear abnormalities. These effects would be expected to inhibit nutrient acquisition not only via physical impairment, but also through limited functioning of hypodermal components. Although highly vacuolated, the intestinal cells, as well as the nuclei, largely remained intact, as reported previously for *B. malayi* and *L. carinii* (Franz et al., 1990). It has been reported that BZs have little to no effect on the intestinal cells of filarial nematodes (Franz et al., 1990; Cárdenas et al., 2010). In this study, damage to the intestine was highly variable and did not follow obvious concentration- or time-dependent trends.

Our study confirmed prior observations on the effects of BZs on filarial female gonads and developing embryos (Awadzi et al., 1982; Howells and Delves, 1985; Franz et al., 1990; Cardenas et al., 2010). FLBZ (1 μM) elicited detrimental effects, predominantly vacuolization, in developing embryos of *B. malayi* following 24 h incubation (Fig. 2D and E). BZs inhibit tubulin polymerization, and given the importance of microtubules for spindle formation and cell division, it is not surprising that pathological alterations were observed in rapidly developing embryos, especially in early stages, more than in the surrounding tissues. Disruption of cell division was also reported in ovaries of *Litomosoides chagasfilhoi* (Cárdenas et al., 2010), *Dirofilaria immitis* (Howells and Delves, 1985), and *O. volvulus* (Awadzi et al., 1982).

The type and extent of damage to filarial tissues caused by FLBZ-R is consistent with reports on nematocidal effects of FLBZ-R (Urbizu et al., 2012). Given the high degree of first-pass metabolism of BZs, it is promising that a main FLBZ metabolite has intrinsic activity against filariae.

Evaluation of drug-induced effects on tissues based on histochemical analyses is challenging. The present study addressed this issue by conducting multiple independent experiments to assess morphological alterations with multiple observers. This study also aimed to provide a numeric representation of drug induced effects. Previous studies report observed effects but did not indicate the level of damage, range of damage over population tested or portion of the worms that exhibited such damage. This study employed histology and light microscopy to assess damage, which is a cost-effective tool that gives a better indication of tissue level effects to provide a comprehensive assessment of damage than transmission election microscopy, which was the main method used in previous studies.

The limitations imposed by *in vitro* culture are an additional challenge to evaluating drug-induced damage. The duration of culture of viable microfilariae is relatively short, unless extended slightly through the use of a feeder cell layer (Taylor, 1960; Townson et al., 1986). In this study, we evaluated damage over a period of three days to avoid the use of a feeder cell layer for several reasons. The first was potential uptake of flubendazole by the cells, which could lead to depletion of the drug from the media. Second, mammalian cells can metabolize flubendazole, depleting it from the medium over time. Finally, if the drug is toxic to mammalian cells at the concentrations and durations of exposure employed, it may lead to the release of by-products that could have affected the parasites. Macrofilarial killing by benzimidazoles generally is seen months after treatment (Franz et al., 1990; Zahner and Schares, 1993), a duration for which *in vitro* culture is not feasible, even with the use of feeder cells. While extended culture would be expected to amplify the effects observed in short term culture, the use of feeder cells could complicate the results.

In conclusion, we found a greater detrimental effect of FLBZ on the hypodermis than the intestine. Given that the hypodermis is suggested to have a greater role in nutrient absorption than the intestine, this suggests that FLBZ may play a role in nutrient deprivation of the worm. Furthermore, the current results support previous findings that BZs have detrimental effects on embryonic development. We found that FLBZ damaged tissues required for development and survival following exposure to ≥100 nM for ≥24 h. This study provides preliminary information concerning the concentration–time profile of exposure to FLBZ required to elicit detrimental effects as measured by changes in morphology.

## Figures and Tables

**Table 1:** Tissue damage scores associated with flubendazole exposure assessed by two methods: A. Classical histopathological survey method; B. Individual section scoring method. Scores are averages from two independent experiments.

Treatment	Hypodermis			Intestine			Early embryos			Late embryos		
	24	48	72	24	48	72	24	48	72	24	48	72
*A*												
Control	0.2	0.1	0.3	0.0	0.0	0.1	0.6	0.6	0.2	0.3	0.4	0.1
100 nM	0.9	0.4	1.1	0.3	0.3	0.8	0.8	1.4	0.4	0.6	0.2	0.4
1 μM	0.3	0.7	0.4	0.2	0.3	0.0	0.5	1.3	0.5	0.0	0.0	0.4
10 μM	0.9	0.6	0.9	0.3	0.2	0.1	0.6	0.6	0.9	0.2	0.0	0.2
*B*												
Control	0.5	1.2	0.6	0.0	0.2	0.3	0.9	1.1	0.0	0.2	0.2	0.7
100 nM	1.6	1.5	1.6	1.5	0.2	1.8	1.4	1.9	1.3	0.8	0.4	0.6
1 μM	1.6	1.6	2.1	0.4	1.2	0.4	1.3	1.9	1.3	0.0	1.3	1.1
10 μM	2.0	1.8	2.0	1.1	1.8	0.7	1.6	1.6	2.2	1.5	1.3	1.3

**Table 2:** Tissue damage scores associated with reduced flubendazole exposure assessed by two methods: A. Classical histopathological survey method; B. Individual section scoring method. Scores are averages from two independent experiments.

Treatment	Hypodermis			Intestine			Early embryos			Late embryos		
	24	48	72	24	48	72	24	48	72	24	48	72
*A*												
Control	0.6	0.5	0.0	0.0	0.0	0.0	0.5	0.3	0.2	0.2	0.0	0.1
100 nM	0.3	0.1	0.0	0.1	0.4	0.0	1.4	0.5	0.9	0.7	0.3	0.1
1 μM	0.5	0.5	0.8	0.0	0.0	0.6	0.8	0.9	1.1	0.1	0.4	0.3
10 μM	0.6	0.6	0.6	0.0	0.1	0.3	0.2	0.6	0.9	0.1	0.0	0.5
*B*												
Control	0.9	0.8	0.3	0.0	0.2	0.1	0.7	0.8	0.1	0.1	0.3	0.0
100 nM	1.1	0.9	0.9	0.4	0.4	0.6	1.7	0.6	1.2	1.4	0.2	0.0
1 μM	1.4	1.0	1.8	0.3	0.3	1.3	1.4	0.9	1.6	0.5	0.3	0.2
10 μM	1.2	1.4	1.9	0.2	0.6	0.8	0.8	1.1	1.5	0.2	0.3	0.6
